# Evaluating diabetes and hypertension disease causality using mouse phenotypes

**DOI:** 10.1186/1752-0509-4-97

**Published:** 2010-07-20

**Authors:** Hong Yu, Jialiang Huang, Nan Qiao, Christopher D Green, Jing-Dong J Han

**Affiliations:** 1 Chinese Academy of Sciences Key Laboratory of Molecular Developmental Biology, Center for Molecular Systems Biology, Institute of Genetics and Developmental Biology, Chinese Academy of Sciences, Lincui East Road, Beijing, 100101, China

## Abstract

**Background:**

Genome-wide association studies (GWAS) have found hundreds of single nucleotide polymorphisms (SNPs) associated with common diseases. However, it is largely unknown what genes linked with the SNPs actually implicate disease causality. A definitive proof for disease causality can be demonstration of disease-like phenotypes through genetic perturbation of the genes or alleles, which is obviously a daunting task for complex diseases where only mammalian models can be used.

**Results:**

Here we tapped the rich resource of mouse phenotype data and developed a method to quantify the probability that a gene perturbation causes the phenotypes of a disease. Using type II diabetes (T2D) and hypertension (HT) as study cases, we found that the genes, when perturbed, having high probability to cause T2D and HT phenotypes tend to be hubs in the interactome networks and are enriched for signaling pathways regulating metabolism but not metabolic pathways, even though the genes in these metabolic pathways are often the most significantly changed in expression levels in these diseases.

**Conclusions:**

Compared to human genetic disease-based predictions, our mouse phenotype based predictors greatly increased the coverage while keeping a similarly high specificity. The disease phenotype probabilities given by our approach can be used to evaluate the likelihood of disease causality of disease-associated genes and genes surrounding disease-associated SNPs.

## Background

Common complex diseases, such as diabetes, cardiovascular diseases, hypertension and cancers, have strong genetic components, but their genetic risk loci are difficult to identify reliably until the recent development of array-based genotyping technology. Wellcome Trust Case Control Consortium (WTCCC) [1] and others have used microarrays of commonly occurring single nucleotide polymorphisms (SNPs) to map genome-wide associations of SNP loci to common diseases and identified hundreds of association loci. However, this technology was designed to efficiently cover common genetic variations and was not designed to test rare SNPs or coding polymorphisms. In only a few cases were coding polymorphisms identified, suggesting that SNPs were only associated and not causative. Assignment of the nearest genes to these association signals as the associated genes, although a common practice, has been found to be not reliable [2]. The disease-associated genes responsible for the SNP association signals can be far away from the SNPs and are not readily mapped [3,4]. Therefore, what genes and how they are responsible for the association signals remain an urgent post-GWAS issue.

Although many network-based ranking strategies have been developed [5-8], these approaches can only implicate genes that are more functionally associated with the disease genes, but not disease causal genes. Another major drawback of these methods is that they are greatly influenced by the overrepresentation of "hot genes" that are much more studied than other genes, leading to a biased evaluation. Therefore, an unbiased evaluation method for disease causality of a gene is still lacking. The ultimate proof that a gene or locus is causative to a disease comes from replicating disease phenotypes in a genetic model of the gene or allele. Human genetic mutation and phenotypes have been well curated in the Online Mendelian Inheritance in Man (OMIM) database [9] and have been used to evaluate phenotype similarities between different gene perturbations [10]. However, the coverage of the human genetic disease phenotypes is very limited (only 3,259 genes are covered by OMIM and the great majority of them are related to monogenic diseases). In contrast, a plethora of mouse genetic phenotypes are available but have never been systematically examined before. The Mouse Genome Informatics (MGI) database [11] contains phenotypic descriptions based on the controlled terms in 'phenotype ontology' (PO) for mutants of 12,302 genes (5,667 of which can be directly mapped to human Entrez genes). Moreover, the numbers of both genes and phenotypes in MGI are growing rapidly.

Although not all genes in MGI are tested for all the phenotypes, the appearance of partial or similar phenotypes to a disease often implicate the existence of other phenotypes of the disease. Complex diseases, such as diabetes often display multiple co-appearing clinical traits (phenotypes), which provide a better chance to determine whether a gene perturbation may cause such diseases than for simple genetic diseases consisting of only one or two phenotypes. We therefore took advantage of the well-organized tree-like structure of PO in the MGI database and developed a decision tree-based classifier to quantify, given the observed phenotypes, the likelihood (expressed as weighted probabilities) that perturbation of a single gene would cause the common metabolic diseases hypertension (HT) or type 2 diabetes (T2D) phenotypes (Methods). We show that the phenotype probabilities given by our classifier can be used to uncover the biological processes preferentially targeted by these common metabolic diseases and to evaluate the likelihood of disease causality of genes linked to GWAS signals.

## Results

### Disease phenotype classifier

**Figure 1:**
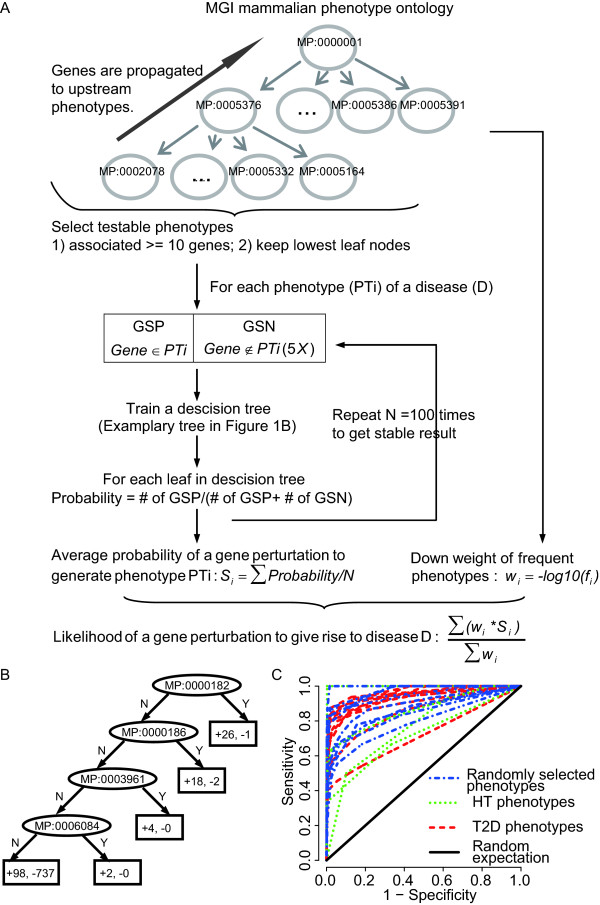
**Flow chart of scoring each gene perturbation's probability of causing disease phenotypes**. (**A**) Decision tree-based phenotype classifiers give the raw probability scores based on expanded and filtered phenotype descriptions (Phenotype Ontology terms) averaged over 100 trees using different random negative gene sets, penalized by the phenotype commonality, and finally summed over all the phenotypes for a disease. See text and Additional File 1 for details. (**B**) An exemplary decision tree for the phenotype "MP00001556" learned from the MGI data. Starting from the top root node, if one gene is annotated with the phenotype in each node (ellipse), it travels down to the branch of "Y"; otherwise to the branch of "N". Leaf nodes (rectangle) represents the number of GSP(+) and GSN(-) in training set that located in this leaf node through all the splits shown in its parent nodes. (**C**) ROCs of 10-fold cross-validation for decision tree-based phenotype classifiers on 10 randomly selected phenotypes (blue dot-dashed lines), HT phenotypes (green dotted lines) and T2D phenotypes (red dashed lines). The black solid line indicates random expectation. Sensitivity = TP/(TP+FN) and false positive prediction rate (1-specificity = FP/(FP+TN)) were used as the y-axis and x-axis variables, where TPs (true positives) are positive predictions which belong to gold standard positives (GSPs), FNs (false negatives) are negative predictions which belong to GSPs.

As illustrated in Fig. 1A and 1B, we first trained decision trees to assign a probability score of whether a gene perturbation causes a phenotype. For each target phenotype *PT_t _*which is associated with a set of *GSP_t _*and one of the 100 randomly selected *GSN_t _*gene sets, we train a decision tree using the presence or absence of all other testable phenotypes (Methods) to assign a probability value of whether a gene has the phenotype *PT_t_*. The training was done using the C4.5 algorithm with details described in Methods. In the end each leaf node represents the probability of a gene to be associated with *PT_t _*given the gene has or does not have all the phenotypes in its parent nodes, in other words, all the nodes it traversed from the top node of the tree (Fig. 1B).

Then to predict and calculate the probability for a new test gene to have the phenotype, we search from the top of the decision tree to locate the node by matching all of its other phenotypes to the nodes in the tree and assign the (GSP/GSP+GSN) value associated with the node to the gene. Thus, genes traced to different nodes will be assigned different probabilities. Then a weight was introduced to correct (by punishing) the abundance of different phenotypes. The sum of weighted probabilities of a gene to cause all the different phenotypes of the disease was assigned to the gene to measure the likelihood of a gene perturbation to result in HT or T2D phenotypes (Fig. 1A, Methods). In order to evaluate the prediction accuracy first at the phenotype level, we examine the cross-validation results of the decision trees based on 10 randomly selected phenotypes and all the phenotypes associated with T2D and HT (Fig. 1C). The ROCs in the cross-validations all have area under curve (AUC) between 0.717 to 0.999 as compared with the randomly expected AUC of 0.5, indicative of high sensitivity and specificity of the decision trees in predicting the phenotypes (Fig. 1C).

### Phenotype scores reflect biological pathways perturbed in HT or T2D

**Figure 2:**
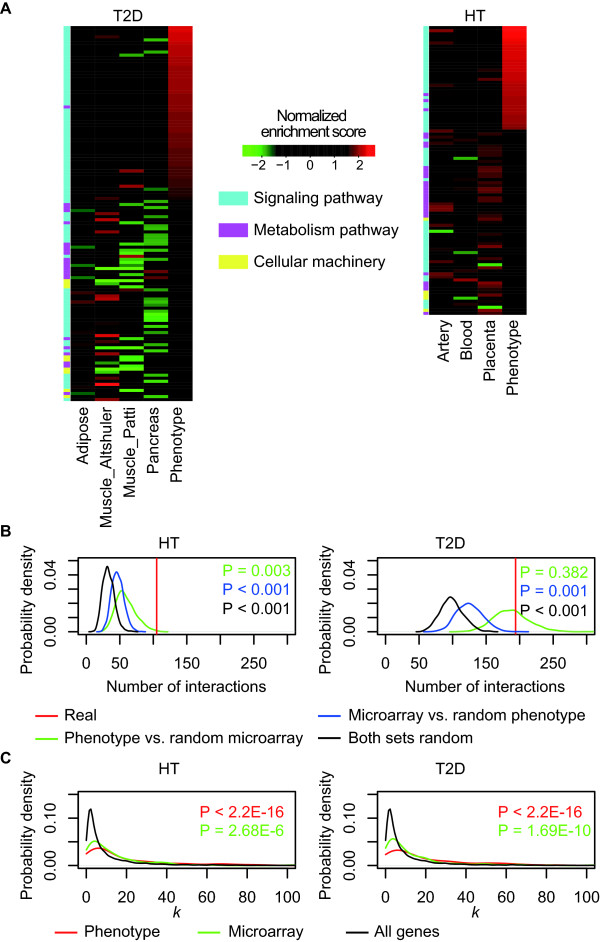
**Pathways enriched among genes with high HT or T2D phenotype probabilities or differentially expressed genes**. (**A**) Enriched pathways are listed according to their HT and T2D phenotype probabilities. The color in the heat plot indicates the normalized enrichment score given by the GSEA program. A black cell indicates that the pathway is not significantly changed in the analysis. For microarray expression analysis, the significantly up-regulated and down-regulated pathways are colored red and green, respectively. (**B**) Number of links between genes with high phenotype probabilities (> 95% specificity as determined in Fig. 4.) and those differentially expressed (RankProd pfp < 0.01) and its significance determined by Monte Carlo simulations. The red line indicates the number of interactions between the genes that have high phenotype probabilities (gene set A) and the genes only differentially expressed in various tissues combined (gene set B) as detected by microarray experiments. The black, blue or green curves indicate the probability density distributions of the number of links if genes in both set A and set B, either A (blue curve) or B (green curve) are replaced by randomly selected genes. Empirical *P *values are labeled on each graph. (**C**) Distribution of interaction degree *k *of high disease phenotype probability (> 95% specificity) genes (red curve), differentially expressed genes (green curve) and all genes in the interactome network (black curve). The Wilcoxon rank test *P *values of increase in k compared to the background 'all genes' are indicated for each gene set.

Using the Gene set enrichment analysis (GSEA) software [12] to test for enriched pathways annotated in the GSEA molecular signature database [12](Methods), we found that the genes having high probability to cause diabetes and hypertension phenotypes are enriched for signaling pathways regulating carbohydrate, fat and other aspects of energy metabolism but not the metabolic pathways themselves (*P *< 0.0001 after multiple testing correction, Fig. 2A, Additional File 1, Fig. S1). This is in sharp contrast to the situation encountered when analyzing the gene expression data for T2D, where mostly metabolic pathways, such as the oxidative phosphorylation and fat metabolism, have been found to be the most significantly differentially expressed, but not regulatory pathways, such as the insulin pathway, despite abundant evidence supporting their role in diabetes [13,14]. To confirm our result was not due to the bias in the GSEA molecular signature database or the GSEA program, we applied the same method to the differentially expressed genes between control and diabetic cases (Methods). Indeed, we also found that metabolic pathways are the most significantly changed in expression levels in muscle, pancreas or adipose tissue in T2D cases (Fig. 2A, Additional File 1, Fig. S1). We also found a similar situation for HT (Fig. 2A, Additional File 1, Fig. S1). Accordingly, only marginal overlaps exist between the genes with high HT or T2D phenotype probabilities (> 95% specificity, see below) and the genes significantly differentially expressed between cases and controls (RankProd pfp < 0.01) (Additional File 1, Fig. S1). These are similar to a previous observation that genes identified in genetic screens are enriched for regulatory pathways, whereas differentially expressed genes identified by microarray analysis are enriched for metabolic pathways [15]. Through network and experimental analysis, Yeger-Lotem *et al*. found that the genetic screens usually identify regulators and are critical for the phenotypes whereas the differentially expressed genes are modulated by these regulators and are indirect reflections of genetic changes in the regulatory network [15].

If this is also true in our case, we would expect genes with high HT or T2D phenotype probabilities to be more significantly linked to the metabolic pathways found through gene expression analysis than random expectation. To test this, we examined the interactions between these two sets of genes using the annotated functional interactions curated in the Human Protein Reference Database (HPRD) and KEGG databases. We measured the number of interactions between the two sets of genes. Indeed, the two sets of genes were significantly linked than randomly expected as shown by Monte Carlo simulations (Fig. 2B, Additional File 1, Fig. S2B). These results suggest that these complex diseases are caused by dysregulation of metabolism rather than metabolism *per se*.

Genes with high interaction degrees (*k*, number of links) or hubs in the interactome networks often play critical regulatory functions and are more likely to be disease-associated [16]. Meanwhile disease-related genes generally have higher degrees than non-disease related genes [16]. Consistent with these previous findings, the genes with high HT or T2D phenotype probabilities (> 95% specificity, see below) also have significantly higher interaction degrees (average *k *= 21.6 and 23.9 for HT and T2D) than differentially expressed genes (average *k *= 16.8 and 15.0 for HT and T2D), which have slightly higher degrees than the average genes in the interactome network (average *k *= 11.5) (Fig. 2C). This suggests that phenotype probabilities given by our predictors are indeed more likely to identify disease causal genes than differential expression analysis.

### Evaluating various disease-association datasets for disease causality

To see if the phenotype probabilities can serve as an unbiased benchmark for evaluating various disease-association datasets, we examined the phenotype probabilities of a few well-known collections of HT and T2D-associated genes. The Online Mendelian Inheritance in Man (OMIM) database and the Gene Association Database (GAD) list many genes that have been found to be associated with these diseases. However, some of the genes had been selected by a candidate gene approach and hence might be biased toward genes functionally related to certain biological processes. Moreover, some of the associations have been found in small sample sets and have not been replicated in an independent study. On the other hand, the GWAS signals are functionally unbiased but have largely not been attributed to causal or functional variants in genes. To another extreme, the KEGG database has annotated T2D pathways based on molecular functions.

**Figure 3:**
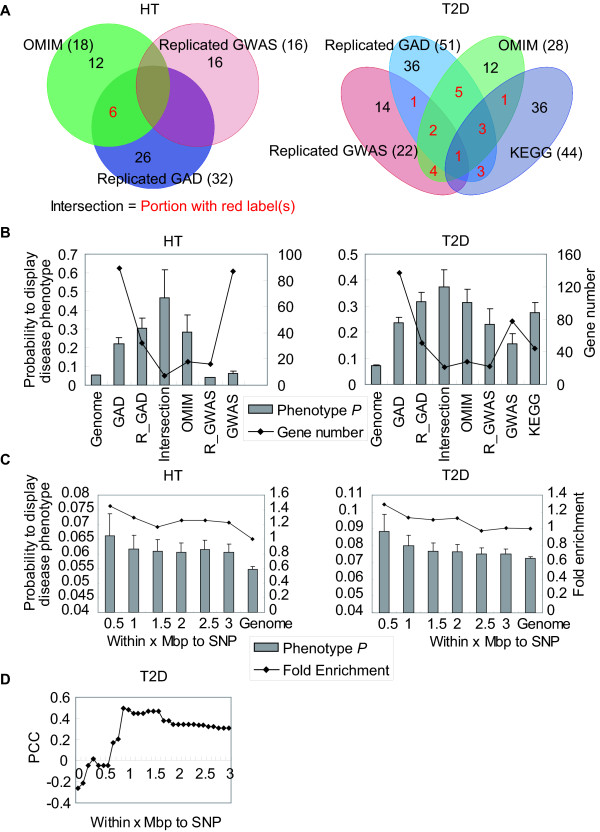
**Evaluation of different HT- and T2D-associated gene sets to cause disease phenotypes when perturbed**. (**A**) The Venn diagrams of different HT- and T2D-associated gene sets. 'GWAS' refers genes nearest to GWAS SNPs. 'R_GAD' are genes in GAD that have multiple references. 'R_GWAS' are replicated GWAS-identified genes as summarized in ref. [1,17-24]. (**B**) Average probability of gene perturbation to cause disease phenotype for different gene sets. The mean and standard errors in each group are marked by the height of the bars and the length of the whiskers. Number of genes within each gene set is indicated by the connected line with units labeled at the right y-axis. (**C**) Average phenotype probabilities (bars scaled to the left y-axis) and fold enrichment for the genes with high phenotype probabilities (> 95% specificity, connected line scaled to the right y-axis) inversely correlate with the distance to the genomic locations of GWAS SNPs. (**D**) T2D phenotype probabilities of genes (the maximal within the x Mbp of SNPs as indicated by the x-axis) correlate with T2D association odds ratio (OR) of the nearby GWAS SNPs.

We found that, as expected, genes listed by multiple sources as disease-associated (the 'Intersection' genes, Methods, Fig. 3A and Additional File 2) are the most likely to cause disease-like phenotypes upon perturbation, much more likely than the unfiltered genes in GAD or OMIM, according to the disease phenotype probabilities predicted from the MGI phenotypes (Fig. 3A and 3B). Although KEGG genes are the most functionally connected genes, they are much less likely to contribute to the disease phenotypes than the 'Intersection'. In contrast, the genes nearest to the authentic association signals identified by GWAS [1,17-24], do not necessarily contribute to T2D phenotypes as shown by the lowest HT- or T2D-phenotype probabilities compared to other datasets (Fig. 3B). However, in both GWAS and GAD datasets the genes replicated in more than one study (R_GWAS and R_GAD) give higher phenotype probabilities than the unreplicated ones, except R_GWAS for HT (Fig. 3B).

Furthermore, both the average phenotype probabilities and the fold enriched over background (Methods) for the genes with high HT or T2D phenotype probabilities (> 95% specificity, see below) increase as the distance gets closer to the genomic locations of GWAS SNPs (Fig. 3C). In addition, the maximal T2D phenotype probabilities among genes within ± 1 Mbp of T2D-associated SNPs are significantly correlated with the case versus control odds ratios (ORs) of the SNPs (Pearson correlation coefficient = 0.480, linear regression slope *P *= 0.03, Fig. 3D). In fact, the correlation can be observed in a rather broad region surrounding the disease-associated SNPs. While not correlated within 0.5 Mbp of the SNP, it reaches the highest level around 0.9 Mbp (Fig. 3D). These results confirm that phenotypic probabilities predicted from MGI phenotypes can indeed serve as an unbiased benchmark for the quality of association signals, and suggest that they may also be used as indicators for disease causality evaluation for genes linked to the disease-associated SNPs.

### Predicting HT or T2D causal genes for GWAS signals

**Figure 4:**
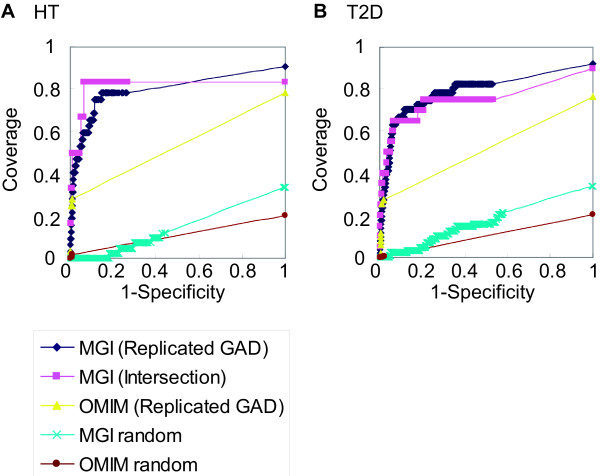
**Performance of phenotype scores in predicting HT- and T2D-associated disease causal genes**. ROCs for HT (**A**) and T2D (**B**). Replicated GAD genes or 'Intersection' genes shown in Fig. 3A were used as GSPs, and all the other genes were treated as GSNs to score all human genes using MGI or OMIM-phenotype-based predictors. For a given phenotype predictor, each gene in the genome was rank-ordered by the phenotype scores. Then at various rank cutoffs, the coverage, or the percentage of the total gold standard positives (GSPs) recovered, and the false positive prediction rate (1-specificity = FP/(FP+TN)) were calculated and plotted as the y- and x-axis variables on an ROC. FPs (false positives) are positive predictions which belong to gold standard negatives (GSNs). TNs (true negatives) are negative predictions which belong to GSNs. For control ROCs, the GSPs were replaced by the same number of randomly selected genes from the total 16023 genes we probed in this study.

To test the possibility of using phenotype probabilities to predict disease causal genes linked to GWAS SNPs, we need a Gold Standard Positive (GSP) dataset to quantify its performance. As shown above, among various sets of HT- or T2D-associated genes, the 'Intersection' genes are mostly likely to cause diseases, followed by a larger gene set of replicated GAD genes (Fig. 3A and 3B, Methods). Using either one of these datasets (Additional File 2) as GSP genes and all the genes that are not labeled as associated genes in GAD, OMIM or ref. [19,20] as the Gold Standard Negative (GSN) genes, we measured the coverage of the total GSP genes versus the specificities of predicting the GSP genes (Methods). The Receiver Operator Curves (ROCs) of the MGI phenotype probabilities are very similar using these two different GSP datasets (Fig. 4), indicating the performance evaluation results are rather robust against variations in the choice of GSP gene sets, and that the similar ROCs might be extrapolated if the GSP genes include only HT or T2D causal genes.

We also compared the prediction power of the MGI (mouse) phenotype probabilities to OMIM (human) phenotype similarities, which are the sum of pair-wise similarity scores between HT/T2D and each of the diseases described for a candidate gene in the OMIM database (Methods). To do this, we used the GSP genes shown in Fig. 3A and treated all the other genes as GSN to plot ROCs by scoring all the human genes using the MGI or OMIM phenotype-base predictors. Because over 80% of the Intersection genes are present in OMIM, using the Intersection genes as GSPs will greatly overestimate the accuracy of the OMIM-based phenotype scoring. Therefore, we only used the replicated GAD gene set to fairly compare MGI phenotype probabilities to OMIM phenotype similarities. The prediction coverage of the MGI phenotype probabilities is obviously much higher than the OMIM phenotype similarities, while their specificities are similar (Fig. 4). The useful coverage of MGI phenotype probabilities is 2.78- and 3.00-fold of that of OMIM phenotype similarities for HT and T2D, respectively (comparing the second to last points on the ROCs in Fig. 4), suggesting it as a valuable resource for phenotype quantification and disease causal gene prediction. The control ROCs, where GSPs were replaced by the same number of randomly selected genes, all appear to be straight diagonal lines from the start point of zero coverage and zero specificity to the end point of zero specificity and maximal coverage for a particular dataset (Fig. 4). This confirms that the phenotype predictors are not biased to give GSPs higher scores, and that it is the phenotypic correlations of disease-associated genes that allow identification of disease genes.

The GWAS by WTCCC has reported association loci at various significance levels. Although the study reported a *P *value < 10^-7 ^as the most confident criteria for disease association, a *P *value < 10^-4 ^might also indicate real association with a relatively higher false discovery rate [1]. Together with the signals that have been identified in other GWAS [17-24], at 95% specificity level, we have predicted 22 and 18 genes mostly likely to be the causal genes for HT and T2D GWAS signals (Additional File 3). These genes are again enriched for signaling pathways regulating metabolism, but not enriched for metabolic pathways (data not shown).

## Discussion

The higher than average phenotype probabilities and the enrichment for high probability genes at the vicinity of GWAS SNPs (Fig. 3C) support the reliability of our phenotype predictors as well as the quality of association data. These were further reinforced by the correlation of the maximal predicted probability to generate T2D phenotypes surrounding T2D-associated SNPs with the OR of the T2D-associated SNPs. The lack of such a correlation for HT might be due to the inhomogeneity of the case populations, as reflected by the drastic differences in allele frequencies: 44% of the HT OR values of are < 1, in which case the major alleles, but not the minor alleles, are associated with the disease, which has never occurred for T2D and most other complex diseases [1]. This may suggest that HT is associated with a broad spectrum of disease etiologies. Thus, in some cases the most common major alleles may cause disease and are detrimental, whereas the minor allele counter-intuitively can offer protection, while in other cases the scenarios are exactly the opposite.

As the more strongly a gene perturbation near a SNP influences the disease phenotype, the higher the SNP's odds ratio (OR), in Fig. 3D we calculated the correlation (PCC) between the maximal phenotype probability of genes within x Mbp of SNPs and the T2D association OR of the nearby GWAS SNP to identify the optimal region where a causal gene is identified for a causal SNP. When x is small, the real disease causal gene is not yet included in the region very close to the SNPs, which leads to the low correlation between phenotype probability and OR as both are at the background level. With the increase of x, some real disease related genes are included, which increase the PCC. The PCC reaches its peak when x = 1, indicating the region of 1 Mbp around the majority of SNPs covered the most (in number and in probability) causal genes. After that, while x is increasing, more false-positive genes are included which either may or may not decrease the correlation depending on whether the highest phenotype probability gene within the region changes, which apparently does not change from 1 Mbp to 3 Mbp and changes only farther than 3 Mbp away.

Many of the genes that we predicted to be the causal genes surrounding the GWAS SNPs have already been shown to be functionally or phenotypically associated with HT or T2D. For T2D, these genes include LEPR, PPARγ, insulin, WFS1, IDE, PPARα, KCNJ11, AQP2, GHRL and ABCC8 (Additional File 3). Most notably, defects in the insulin gene and the insulin degrading enzyme directly affect insulin signaling [25]. The leptin receptor (LEPR) and ghrelin (GHRL) genes balance the regulation of food intake and adiposity [26,27], a risk factor for T2D. PPARγ activation promotes adipocyte differentiation and storage of excess circulating carbohydrates as triacylglyceride [28]. Additionally, KCNJ11 and ABCC8 form the subunits for the ATP-sensitive potassium channel that is required for glucose-stimulated insulin secretion from pancreatic β-cells [29].

Only five out of the 18 predicted T2D causal genes are not found to be co-cited with diabetes by automated co-citation search in the PubMed abstracts as described in [30] (Additional File 3). However, full gene name searches show all genes have functionally relevant roles in diabetes. Loss of histamine receptor H1 (HRH1) impairs leptin control of food intake, leading to obesity [31]. PPARγ-mediated differentiation is directly repressed by the transcriptional modulator WWTR1 [32], whereas PPARγ-mediated lipid storage is indirectly affected by loss of acetyl-CoA carboxylase 1 (ACACA) or mitochondrial glycerol-3-phosphate acyltransferase (GPAM). Although the mechanism is unknown, mutations in the SOX4 gene result in diminished glucose-stimulated insulin secretion [33]. Together, these findings for T2D causal genes further demonstrate the reliability and significance of this methodology.

We focused on hypertension and diabetes due to their commonality, the relatively well-defined phenotypic descriptions of the diseases, and the sufficient number of known disease-associated genes. Conceivably, the methods described here will be applicable to other diseases, given well-defined phenotypic descriptions and a large enough validated gene set for the diseases.

## Conclusions

Despite the enormous advances on GWAS of common disease susceptibility loci, determining causal genetic loci is still a pressing issue to address. We for the first time tapped the rich resource of mouse phenotype data to quantify the probability of gene perturbation to induce phenotypes of a common disease. Our phenotype predictors were indeed able to identify the important regulatory pathways whose deregulation may lead to these metabolic diseases, instead of genes or pathways simply associated with or changed by the diseases. This type of causality inference is a unique feature of genes identified by genetic perturbation and phenotypic analysis and can only be indirectly reflected to a certain degree by some other type of analysis, such as gene-expression analysis. Therefore, genetic perturbation leading to phenotype alteration indeed can serve as a general rule for disease/phenotype causality evaluation. Furthermore, our introduction of mouse phenotype as disease causal effects evaluation criteria and developing it as quantitative criteria allows objective evaluation of various association datasets, and the disease phenotype probabilities given by our approach can be used to evaluate the likelihood of disease causality of disease-associated genes and genes surrounding disease-associated SNPs.

## Methods

An ethics statement is not required for this work.

### Datasets

Phenotypes of mouse gene knock-out or transgenic mutants, together with the mouse gene to human ortholog mapping were downloaded from the Mouse Genome Information (MGI) database http://www.informatics.jax.org/ on Oct. 20, 2009. OMIM data was downloaded from http://www.ncbi.nlm.nih.gov/omim/ on March 24, 2009.

The HT and T2D associated entries in the genetic association database (GAD) [34]http://geneticassociationdb.nih.gov/ were downloaded on Dec 27, 2007. We kept only the genes that have the value 'Y' or 'P' for the attribute 'associated to disease'. This resulted in 89 and 138 genes for HT and T2D, respectively.

We downloaded the HT- or T2D-associated SNPs from the WTCCC website [1] and selected all the SNPs with association *P *< 0.0001. Other GWAS datasets on HT or T2D were obtained from individual GWAS publications [17-24]. SNP signals that have been replicated in multiple large-scale association or GWAS were obtained from ref. [19,20].

Gene expression microarray datasets were obtained from GSE8051, GSE703, and GSE4707 for HT, GSE16415 and ref. [13,14,35] for T2D.

### Compilation of HT and T2D phenotypes

HT and T2D phenotypes were manually selected and mapped to the phenotype ontology terms in MGI based on diagnosis descriptions on Wikipedia and literature [36,37] as follows.

For T2D:

MP:0000182 increased circulating LDL cholesterol level

MP:0000231 hypertension

MP:0001261 obese

MP:0001433 polyphagia

MP:0001556 increased circulating HDL cholesterol level

MP:0001559 hyperglycemia

MP:0001759 increased urine glucose level

MP:0001762 polyuria

MP:0002079 increased circulating insulin level

MP:0005293 impaired glucose tolerance

For HT:

MP:0001776 abnormal circulating sodium level

MP:0004217 salt-sensitive hypertension

MP:0006143 increased diastolic blood pressure

MP:0006144 increased systolic blood pressure

### Training decision trees to score probability of exhibiting disease phenotypes by mouse mutants

In the phenotype ontology tree, all the other leaf node phenotypes that are not phenotype *PT_i _*or a child of *PT_i _*were used as classification attributes in the decision tree to predict the probability of a gene's perturbation to give phenotype *PT_i_*. We used the Weka J48 classifier http://www.cs.waikato.ac.nz/ml/weka/ which implements the C4.5 algorithm to build decision trees. To train a decision tree for target phenotype *PT_i_*, the algorithm starts with all genes in the training set in a single root node and then recursively splits each node N by testing for the presence or absence of the phenotype k that gives rise to the maximal information gain, which is defined as *H*(*N*)*-H*(*N0*)*-H*(*N1*) when splitting N into N0 and N1 by judging whether gene g has been annotated with phenotype k. Here, *H*(*N*) is the entropy of genes at node N, defined as -*PGSP*(*logPGSP*)-(*1*-*PGSP*)*log(*1*-*PGSP*), where *PGSP *is the percentage of GSP genes at node N. When no test at a node N gives a positive information gain, the node is not further split and becomes a leaf node with a probability value associated with it. For each disease phenotype (*PT_i_*), we used the genes associated with *PT_i _*as positive training data and randomly chosen genes (five times the number of the positive instances) that are not associated with *PT_i _*as negative training data. Due to the unevenness of the number of genes associated at different levels of the phenotype ontology tree, we selected the lowest-level phenotypes with > = 10 genes as testable phenotypes to ensure enough training cases and branch points for the decision tree (using > = 20, 30 or 40 genes yielded similar results, Additional File 1). We repeated the negative set selection and decision tree training 100 times and used the average probability given by the 100 decision trees as the final probability for a gene to have the phenotype *PT_i_*. Our settings resulted in a range of 5 to 15 phenotype nodes per decision tree used for each phenotype prediction.

### Likelihood of a gene perturbation to result in the phenotypes of a disease

We trained decision trees to assign a probability (the proportion of true positives (TP) in the leaf of a decision tree) of whether a gene in MGI phenotype database has a disease phenotype (see above), inspired by the method described for assigning gene functions [38]. To account for the abundance of different phenotypes, a weight of *-log_10_(f) *of each phenotype is used to adjust the probability, where *f *is the frequency of the phenotype appearing among all the genes. The sum of weighted probabilities (-∑*log_10_(f)P*) of a gene to all the different phenotypes of the disease is assigned to the gene to measure the likelihood of a gene perturbation to result in phenotypes of a disease. Finally, these summed probabilities were normalized against their possible maximal value within each disease, to maintain their values between 0 - 1.

### Scoring disease similarity

Phenotype similarities between diseases were calculated as previously described [10]. All the OMIM diseases whose description contain the word "hypertension" for HT and "diabetes" or "diabetic" for T2D were used as the collection of reference nodes *R *in the disease similarity network. For a gene *a *in OMIM, its disease phenotype similarity to *R *is defined as *S*(*a*) *= *∑*_i_*∑*_j_*(*s_ij_*), where *i *is a disease associated with *a *and ∉ *R*, disease *j *∈ *R*, and *s_ij _*is the similarity score between diseases *i *and *j*.

### Enrichment of GSEA pathways

Pathways were downloaded from the Gene Set Enrichment Analysis (GSEA) molecular signature database [12] on Oct. 9, 2009. The significance of enrichment was calculated using the Gene Set Enrichment Analysis (GSEA) software [12].

### Differentially expressed genes in microarray experiments

Significantly differentially expressed genes between disease and control groups were determined using the RankProd program [39] at proportion of false positive (pfp) < 0.01 based on log2 fold changes in gene expression over the controls.

### Empirical P values for observed number of interactions

The significance of the observed number of interactions between two sets of genes was determined by an empirical *P *value, which is the frequency for two randomly selected gene sets to have the same or greater number of interactions than what was observed.

### Fold enrichment

Fold enriched over background is defined as (m/n)/(M/N), where M genes out of total N genes are disease related, and within a given gene set with n genes, there are m genes that are disease related.

## Authors' contributions

JDJH conceived and directed the project. YH performed all the analysis except constructing decision tree classifiers and GSEA, which were done by JH and microarray data collection and normalization which were done by NQ, JDJH, HY, JH and CDG wrote the manuscript. All authors read and approved the manuscript.

## Supplementary Material

Additional file 1 **Supplemental Materials for Evaluating diabetes and hypertension disease causality using mouse phenotypes**. Supplemental methods and figures. Click here for file

Additional file 2 **Supplemental Table 1. List of curated HT- and T2D associated gene sets**. List of curated HT- and T2D associated gene sets Click here for file

Additional file 3 **Supplemental Table 2. List of predicted potential causal genes associated HT and T2D GWAS SNPs**. List of predicted potential causal genes associated HT and T2D GWAS SNPs Click here for file
