# Supplementary material for: Evaluating diabetes and hypertension disease causality using mouse phenotypes
Source: BMC Syst Biol. 2010 Jul 20;4:97. doi: 10.1186/1752-0509-4-97 (PMC2917432; doi:10.1186/1752-0509-4-97)

Supplemental Materials for

Evaluating diabetes and hypertension disease causality using mouse phenotypes

Hong Yu[1*], Jialiang Huang[1*], Nan Qiao[1], Christopher D. Green[1] and Jing-Dong J. Han[1, #]

Supplemental Methods

Mapping of SNP genomic coordinates and gene coordinates

Genes surrounding SNPs were mapped using NCBI build 36 coordinates. Refseq hg18 position information was downloaded from UCSC on Dec 21, 2008. Refseq hg18 to NCBI build 36 Entrez gene mapping was downloaded from NCBI on April 21, 2008. After removing 344 Refseq entries that have multiple positions for each sequence and 81 Refseqs that map to multiple Entrez genes, 25,531 unique Refseq entries were mapped to 19,053 Entrez gene IDs with unique positions. When a gene was mapped to multiple Refseqs, if these Refseqs did not completely overlap, then the gene was excluded, otherwise the first start and last stop positions of all the Refseqs for a gene were used as the boundary of the gene. 19,053 genes remained for MGI phenotype scoring.

Minimal number genes used to train a decision tree

To ensure enough training cases and branch points for the decision tree, we selected the lowest-level phenotypes with >= 10 genes (referred to as A10 below). We repeated the decision tree-based phenotype probability calculations for all HT and T2D phenotypes by using the lowest-level phenotypes with >= 20, 30 or 40 genes (referred to as A20, A30 or A40). As measured by the pair-wsie Pearson's correlation coefficient, the final phenotype probabilities predicted from phenotypes of these different levels are

highly similar (below, all *P* values are negligible), suggesting that using >= 10 genes is sufficient to robustly train the decision trees.

HT:

PCC	A10	A20	A30	A40
**A10**	1	0.916509	0.901588	0.841594
**A20**	0.916509	1	0.961271	0.903066
**A30**	0.901588	0.961271	1	0.913323
**A40**	0.841594	0.903066	0.913323	1

T2D:

PCC	A10	A20	A30	A40
**A10**	1	0.98771	0.98376	0.97136
**A20**	0.98771	1	0.98039	0.96863
**A30**	0.98376	0.98039	1	0.9717
**A40**	0.97136	0.96863	0.9717	1

Supplemental Figures

Supplemental Figure 1. Pathways enriched among genes of high HT or T2D phenotype probabilities versus those among genes differentially expressed between controls and cases. This is a scalable, more detailed version of Fig. 2A, with pathway names listed.

Supplemental Figure 2. Overlap and interactions of the genes identified by MGI phenotype probabilities with those identified by differential gene expression.

(**A**) Marginal overlaps between genes of high disease phenotype probabilities (> 95% specificity) and those differentially expressed between cases and controls (RankProd pfp < 0.01). Log2 fold changes are adjusted to [-1, 1] by dividing their maximal absolute values in each disease-control comparison.

(**B**) Number of links between genes of high phenotype probabilities and those

differentially expressed and its significance determined by Monte Carlo simulations. The red line indicates the number of interactions between the genes that have high phenotype probabilities (gene set A) and the genes differentially expressed in different tissues (gene set B) as detected by each microarray experiment (excluding overlapping genes). The curves and the *P* values are similarly derived as in Fig. 2B.

Supplemental Figure 1.

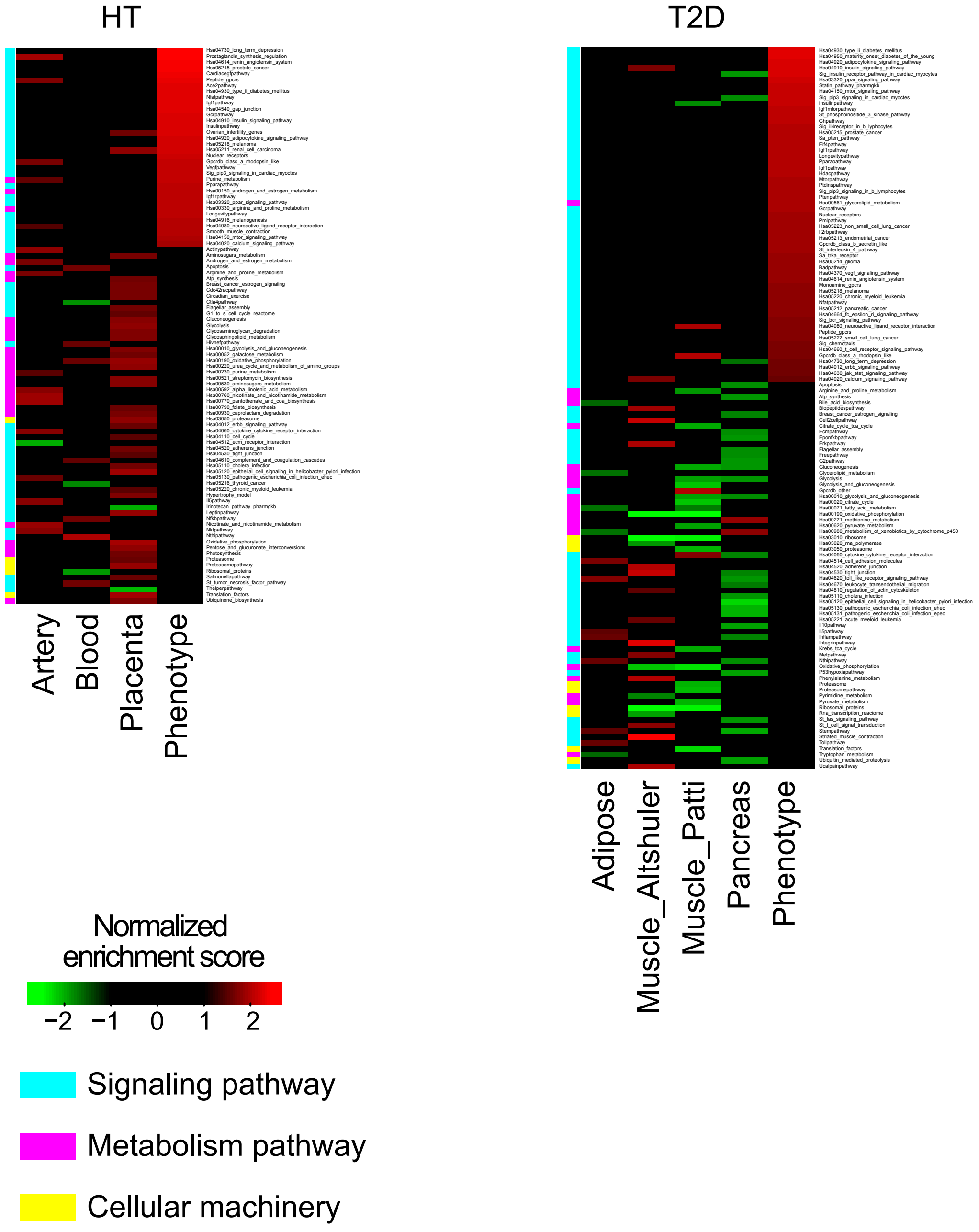

Supplemental Figure 2.

A

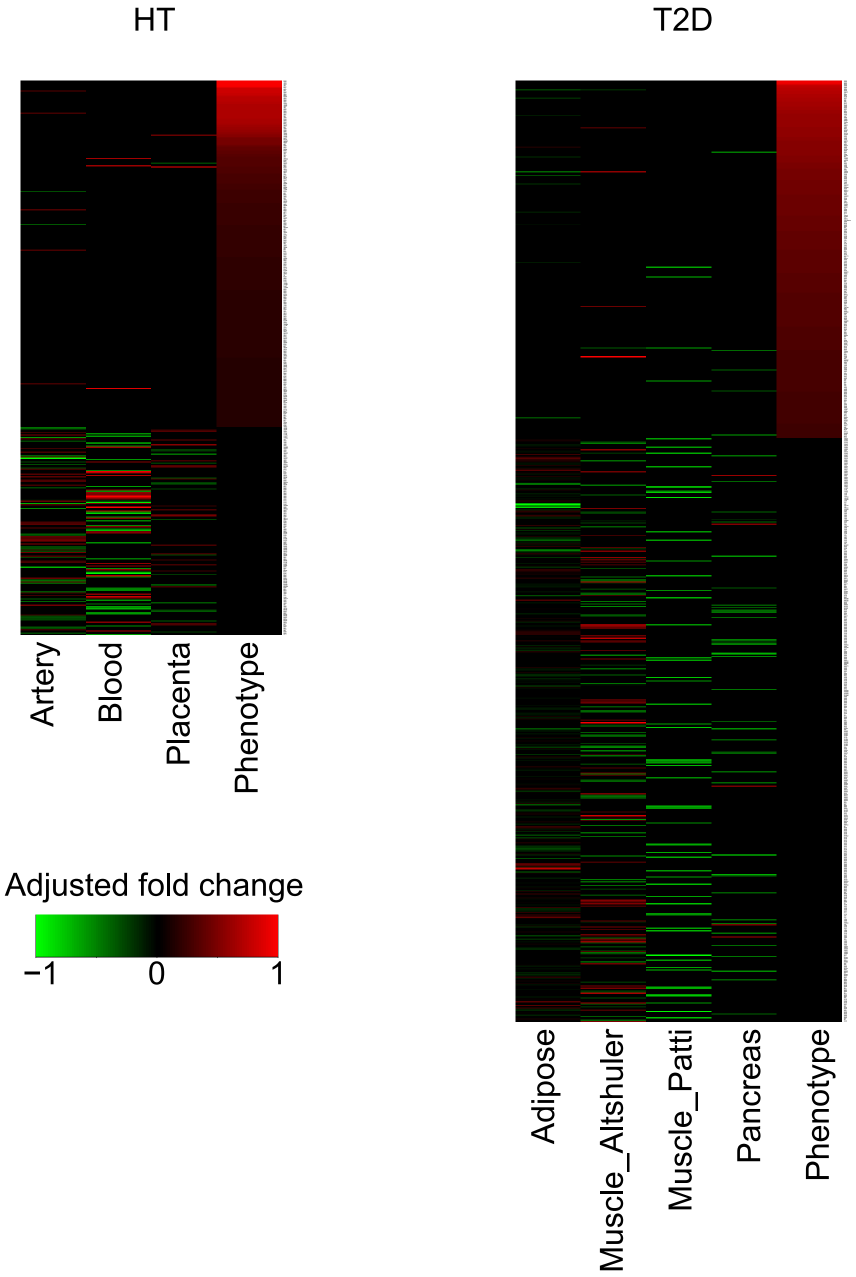

Supplemental Figure 2.

B

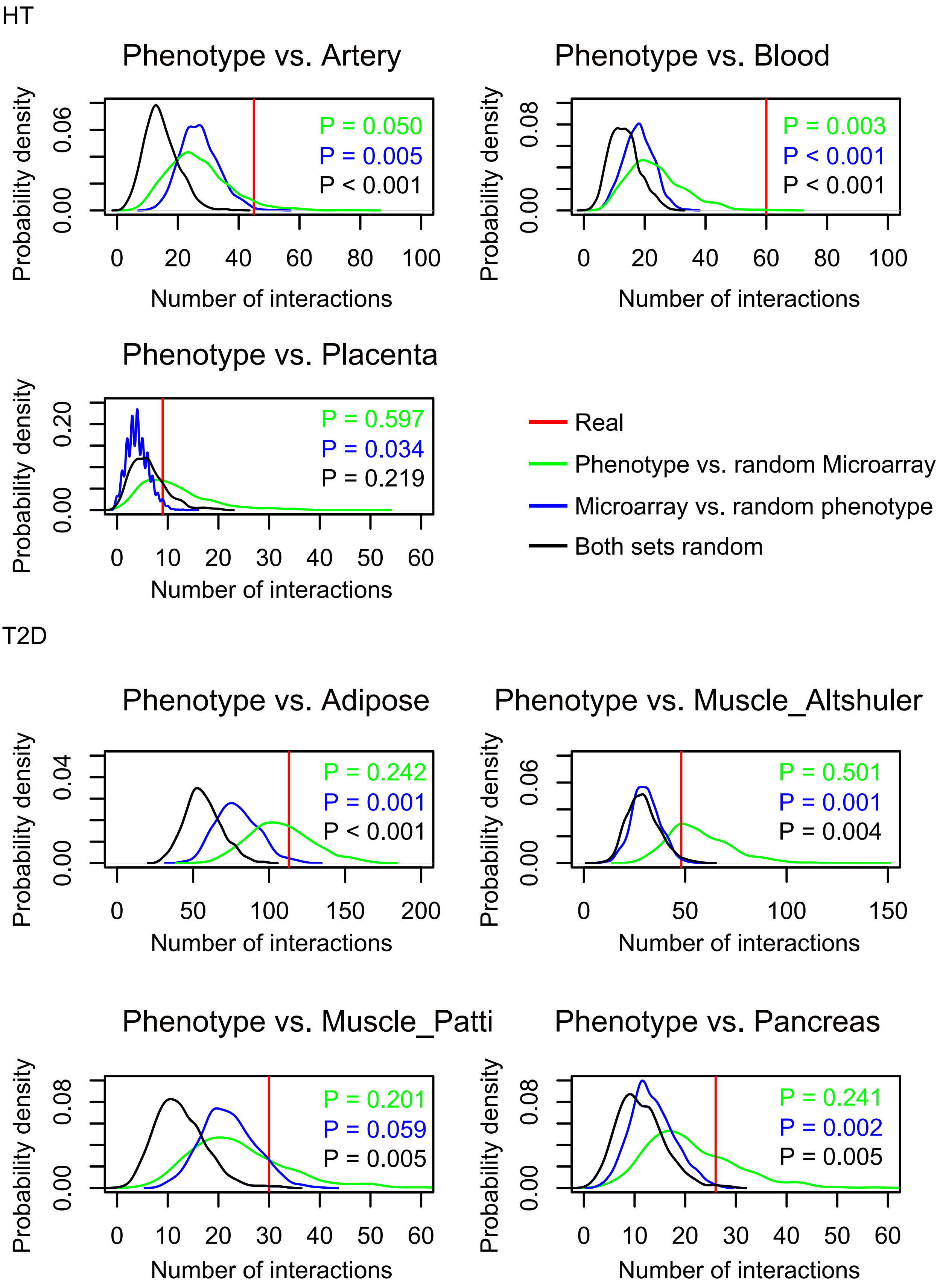

Supplement: Additional file 1 — Supplemental Materials for Evaluating diabetes and hypertension disease causality using mouse phenotypes. Supplemental methods and figures. [file 1752-0509-4-97-S1.PDF]
